# The Age Factor Revisited: Timing in Acquisition Interacts With Age of Onset in Bilingual Acquisition

**DOI:** 10.3389/fpsyg.2018.02732

**Published:** 2019-01-14

**Authors:** Petra Schulz, Angela Grimm

**Affiliations:** Institute for Psycholinguistics and Didactics of German, Goethe University Frankfurt, Frankfurt, Germany

**Keywords:** language dominance, age of onset, timing in monolingual acquisition, language input, bilingualism, early second language acquisition, simultaneous bilingual acquisition, LiSe-DaZ

## Abstract

In this paper, we investigate whether timing in monolingual acquisition interacts with age of onset and input effects in child bilingualism. Six different morpho-syntactic and semantic phenomena acquired early, late or very late are considered, with their timing in L1 acquisition varying between age 3 (subject-verb agreement) and after age 6 (case marking). Data from simultaneous bilingual children (2L1) whose mean age of onset to German was 3 months are compared with data from early second language learners of German (eL2) whose mean age of onset to German was 35 months as well as with data from monolingual children. To explore change over time, children were tested twice at the ages of 4;4 and 5;8 years. The main findings were that 2L1 children had an advantage over their eL2 peers in early acquired phenomena, which disappeared with time, whereas in late acquired phenomena 2L1 and eL2 children did not differ. Moreover, 2L1 children performed like monolingual children in early acquired phenomena but had a disadvantage in the late acquired phenomena with the amount of delay decreasing with time. We conclude that age of onset effects are modulated by effects of timing in monolingual acquisition. Contrary to expectation, input in terms of language dominance, measured as the dominant language used at home, did not affect simultaneous bilingual children’s performance in any of the phenomena. We discuss the implications of our findings for the hypothesis that acquisition of late phenomena is determined by input alone and suggest an alternative concept: the learner’s internal need for time to master a phenomenon, which is determined by its complexity and cross-linguistic robustness.

## Introduction

A growing body of research is devoted to child bilingual language learners (see Chondrogianni, 2018, for an overview). It complements the research on adult second language acquisition by examining age of onset effects among different types of child bilingual acquisition. The goal of our study is to contribute to the debate instigated by Tsimpli (2014) on whether age of onset effects can be modulated by effects of timing in monolingual acquisition. The concept “timing in acquisition” refers to the assumption that L1 development of the phenomena examined in bilingual children systematically modulates other factors such as age of onset and input. To address this issue, data were collected from simultaneous and early successive bilingual children acquiring German as well as from monolingual German children. We asked whether simultaneous and early successive bilingual learners differ regarding early phenomena, with an advantage for the simultaneous bilinguals. We also asked whether (very) late phenomena result in similarly high or low performance, differentiating both groups of bilinguals from monolinguals, as predicted by Tsimpli’s (2014) account. Furthermore, we addressed the issue of how timing in L1 acquisition interacts with differences in amount of language input by investigating the group of simultaneous bilingual children in more detail. This is a group that is often assumed to acquire target languages as their monolingual peers do and within a similar time frame as well (e.g., Genesee and Nicoladis, 2007; Paradis et al., 2011a).

The distinction between simultaneous and successive child bilingual acquisition hinges on the different outcomes postulated by acquisition theories. There is general agreement that acquisition of a second language after age seven qualitatively differs from first language acquisition, reaching the upper cut-off point for a critical or sensitive period for L2 acquisition (see Meisel, 2011, for an overview). At the lower end of the continuum, a consensus prevails that simultaneous acquisition of two languages from birth needs to be considered discretely as well, for this acquisition context falls within the sensitive or critical periods for language acquisition (e.g., Locke, 1997). Regarding the definition of successive childhood bilingualism there is less agreement. Some studies propose that age of onset to the second language occurs between the ages of 1 and 3 years (e.g., Unsworth, 2013a), while other studies suggest that age four constitutes an important cut-off point (Schwartz, 2004; Rothweiler, 2007; Meisel, 2009; Schulz and Tracy, 2011; Unsworth, 2016). Consequently, the upper age limit for what counts as simultaneous language acquisition varies considerably, ranging from birth to about 2 years (De Houwer, 2009). In addition to these theoretically inspired questions, age of onset issues are confounded with country-specific educational practices. This is because the age of onset to the second language often coincides with the age at which children tend to start daycare, where in general the country’s majority language is spoken. In the present study, we use the term “bilingual acquisition” to refer to children who acquire two languages. Following our previous work (Schulz and Tracy, 2011; Grimm and Schulz, 2014a, 2016) the term “early second language acquisition” (henceforth also: eL2) is used to refer to children whose age of onset to the L2 is between the ages of 2;0 and 4;0 years; and the term “simultaneous bilingual language acquisition” (henceforth also: 2L1) is used to refer to children who are first exposed to the “other” language between birth and the age of 23 months. This way we capture the fact that children who are exposed to the second language after the age of 24 months have already developed substantial lexical and grammatical knowledge in their first language and cannot be considered “simultaneous learners” anymore.

In the current study we investigate language domains in morpho-syntax and semantics that comprise early and (very) late acquired phenomena in three different acquisition types. Although these acquisition types could all be called “early,” they differ regarding age of onset and regarding input in a second language. More specifically, we examine whether 2L1 children are like eL2 children. Both acquire German as one of two languages but differ in their age of onset. And we examine whether 2L1 children are like monolingual German-speaking children. Both have comparable ages of onset to German but have different amounts of input in the L2 German. To take into account the interaction of age of onset, timing in L1 acquisition and time of testing we adopted a longitudinal design in which data were collected in two test rounds at ages 4;4 and 5;8, about 16 months apart. To explore whether language dominance in the group of simultaneous bilingual children affects their performance, we determined subgroups of German-dominant, non-German-dominant and balanced simultaneous bilinguals, based on the dominant language used at home. Data on all phenomena were collected with the standardized test LiSe-DaZ (Schulz and Tracy, 2011). In short, our goal was to investigate the effects of age of onset (from birth, around age 3) and of timing in L1 acquisition (early, late, very late) in child bilingualism. Second, we explored whether language dominance, defined as the dominant language used at home, affects simultaneous bilingual children’s performance.

## Factors Influencing Child Bilingual Acquisition

Compared to monolingual children, bilingual children are subject to many more sources of variation in their language environment that may in principle influence their pace and path of acquisition. Factors such as general cognitive abilities and parental socio-economic status play a role for some facets of children’s language development. Additionally two sets of factors are especially relevant in the bilingual language acquisition context (Paradis and Jia, 2017; Chondrogianni, 2018): so-called age factors, related to the age of onset to the second language (see Section “Age of Onset Effects in Child Bilingualism”), and so-called input factors, related to input quantity and quality as well as to language dominance (see Section “Effects of Language Input and Language Dominance”). In some studies length of exposure to the second language has been classified as an input effect as well (e.g., Unsworth, 2016; Chondrogianni, 2018). However, length of exposure is related to age of onset as well as to the child’s chronological age, often resulting in confounds between these factors. We return to this issue in the discussion. Recently, Tsimpli (2014) has proposed “timing in L1 development of the phenomena examined in bilingual children’s performance” as an additional factor (see Section “Timing in L1 Acquisition”). Taking up the well-known observation that results for bilingual children may differ depending on the area of language being investigated, Tsimpli (2014) argues that early and late acquired phenomena result in different outcomes for the different types of bilinguals. According to her account, the linguistic factor “timing in L1 acquisition” needs to be taken into account to meaningfully address the role of age of onset and of language input.

Note that comparison of results in bilingualism studies is sometimes difficult, as studies have investigated different aspects of the acquisition process. Some have focused on the nature of the acquisition path, asking whether the acquisition phases and their sequences are the same and whether the error patterns in each acquisition phase are caused by the same underlying acquisition principles (Meisel, 2009; Schulz, 2013; Unsworth, 2013a; Rothweiler et al., 2017; Schulz and Schwarze, 2017). Others have focused on the pace of acquisition, asking how fast specific acquisition stages are reached and at what age specific structures are mastered (e.g., Grimm and Schulz, 2012; Paradis and Jia, 2017). Still others have focused on the success of acquisition, asking whether successful acquisition, often referred to as native-like attainment, is possible (e.g., Kupisch and Rothman, 2018; see Schulz, 2012, for discussion of native-like attainment in general). Given the standardized nature of our data, our study focuses on questions of pace and success.

### Age of Onset Effects in Child Bilingualism

The presence of age effects in second language acquisition is uncontroversial. A notably robust finding is that child second language learners in general outperform adult second language learners (see the influential study by Johnson and Newport, 1989). This effect has been attributed to the existence of one or several critical periods (Locke, 1997; Meisel, 2009, 2013; see Birdsong, 2006, for an overview) as well as to other cognitive factors (e.g., Klein, 1996; Bialystok and Hakuta, 2010). Recently bilingualism research has started to address the role of age of onset within childhood bilingualism.

One line of research focuses on successive bilingual learners with different ages of onset. Many studies on child L2 acquisition (age of onset of 6 to 7 years) have found that child L2 learners perform much like adult learners and very differently from eL2 children. Evidence for parallels between child L2 and adult L2 has been reported for passive in German (Wegener, 1998) and for verb-second and subject-verb agreement in German (Haberzettl, 2005; Rothweiler, 2006; Chilla, 2008). However, in a study on passives in English, Rothman et al. (2016) found that child L2 learners outperformed children with an age of onset of 4 to 5 years which they attributed to so-called conceptual transfer from the L1 to the learners’ L2. Many studies on eL2 acquisition (age of onset of 3 to 4 years) have found parallels between eL2 and monolingual children. For example, eL2 children were reported to perform in a similar fashion to monolingual children on subject-verb agreement and verb-second in German in a number of different studies, producing the same types of error patterns and showing a delay only regarding the age of mastery (Prévost, 2003; Tracy and Thoma, 2009; Tracy and Lemke, 2012; Grimm and Schulz, 2014b; Rothweiler et al., 2017; Schulz and Schwarze, 2017). eL2 children were also found to acquire interpretation of German wh-questions in a similar fashion to monolingual children, showing a delay of about 1 year (Schulz, 2013). In a study of subject-verb agreement and clitic placement in L2 French, however, Meisel (2008) found that eL2 children’s errors were similar to those found in L2 adults.

A further line of childhood bilingualism research focuses on the comparison of simultaneous bilinguals (2L1) with other populations. Studies comparing 2L1 and eL2 children have not found age of onset effects for the phenomena under consideration. In a study of Dutch neuter gender, Unsworth et al. (2014) found that 2L1 and eL2 children behaved alike regarding consistency of gender assignment and agreement. Given their selection of participants, the authors were able to consider length of exposure and age of onset separately and found that target-like gender marking was controlled by length of exposure rather than by age of onset. In a study of the comprehension of German wh-questions in 2L1 children and eL2 children Roesch and Chondrogianni (2016) did not find an effect of age on onset; differences between the groups were accounted for by length of exposure. Similarly, in a study of case and gender marking in noun phrases in German Hopp (2011) found strong correlations between length of exposure and eL2 children’s performance, but no effect of age on onset.

Studies comparing 2L1 children to monolingual children have generally found that simultaneous bilinguals were not disadvantaged, acquiring the two languages in a similar fashion and at a similar pace as their monolinguals peers (for an overview see Genesee and Nicoladis, 2007). Some studies reported specific acceleration effects, whereas other studies reported delays (see Hager and Müller, 2015, for a discussion of robust and non-robust domains in 2L1 children in comparison to monolingual children of the same language; see Müller, 2017, for a discussion of sources for acceleration and delay). Acceleration has been found for the area of morpho-syntax, with functional elements from the more developed language serving as a bootstrap for the acquisition of the functional elements of the other language (e.g., Gawlitzek-Maiwald and Tracy, 1996). In a similar vein, Kupisch (2006) argued for a “booster” effect in the development of determiners, caused by the bilingual children’s ability to use their knowledge in one language when producing determiners in the other language. Delays have been mainly reported for the lexical domain, with a 2L1 child’s vocabulary in either language being smaller than that of monolingual same-age peers and with less accurate performance on rapid lexical retrieval tasks (Bialystok, 2009). But delays have also been found for grammatical gender (see Gathercole and Thomas, 2009, for Welsh; Eichler et al., 2013, for German neuter gender) and for dative case/gender in German (Hager and Müller, 2015).

In short, whereas the existence of age of onset effects in second language acquisition is undisputed, many open questions remain, including which are the relevant cut-off points and whether the slogan “earlier is better” always holds. Also, effects of age of onsets do not occur in isolation; they are related to factors such as length of exposure and the age at which the learners were studied. The latter factor is important because it determines whether we can expect bilingual learners to have had enough time to catch up and acquire the specific phenomenon by the tested age. This in turn points to the factor “timing in L1 acquisition” to be considered next.

### Timing in L1 Acquisition

According to Tsimpli (2014), timing in L1 development of the phenomena examined in bilingual children’s performance interacts with other factors such as age of onset and input. Differentiating between early, late and very late acquired phenomena, Tsimpli proposes that this classification reflects the differing impact of narrow syntax. Early phenomena are core, parametric and narrowly syntactic, whereas late and very late phenomena involving syntax-external or language-external resources are not narrowly syntactic. Put differently, core grammatical properties are products of narrow syntax and exclude semantic effects. Macroparameters such as object verb directionality (OV/VO) and verb-second and their related microparametric options hence constitute the core component of language, which is acquired early. Phenomena that do not belong to the core are associated with components outside of narrow syntax. These late phenomena, acquired after age five, may involve semantics and pragmatics as well as non-verbal cognitive resources. More specifically, they may require knowledge at the semantic-syntactic interface and sensitivity to contextual information as with quantification and exhaustivity in wh-questions or they may require increased computational efforts as for instance in the comprehension of object-questions (Tsimpli, 2014: 293–294). Most importantly for the present study, it is argued “[...] that early phenomena can differentiate between simultaneous and (early) successive bilingualism with an advantage for the former group, while the other two reveal similarly (high or low) performance across bilingual groups, differentiating them from monolinguals” (Tsimpli, 2014: 283–284).

In the following we provide a classification of selected phenomena as early, late or very late, which have been studied in bilingual acquisition and which were tested in the present study (see Schulz, 2007; Schulz and Grimm, 2012, for an overview of the timing of acquisition in monolingual German). In line with Tsimpli (2014) we assume that earliness and lateness of the phenomena depend on whether additional resources or language submodules are involved, but we remain agnostic as to whether early phenomena have to belong to core-syntax. It may be that formal complexity plays a role, i.e., how much idiosyncracy and irregularity is involved in a construction (see Culicover, 2014), which may or not may not align with the distinction between core and non-core (see also the contributions in Newmeyer and Preston, 2014).^1^ Among the early phenomena, acquired before age 5, are object-verb directionality (OV/VO), verb-second and subject-verb agreement in German (Clahsen, 1986; Tracy, 1991) as well as acquisition of subordinate clauses (Bloom et al., 1989). These phenomena belong to the core expressing macroparameters and do not involve semantics. Furthermore, grammatical gender in Greek (Tsimpli, 2003) and telicity (Penner et al., 2003; see Schulz, 2018, for an overview) are acquired early. For gender in Greek it is argued that it shows consistent cues for gender values on nouns making it a grammatical gender language (Tsimpli, 2014: 298). For telicity, it could be argued that it involves mostly lexical knowledge and no resources at the level of sentential semantics. Among the phenomena referred to as late, i.e., acquired around age 5, are passives (Armon-Lotem et al., 2015) and comprehension of relative clauses and wh-questions (Friedmann et al., 2009). According to Tsimpli (2014: 295) these phenomena require additional semantic or lexical information, with the possible exception of relativized minimality accounts of wh-movement (see the discussion in Friedmann et al., 2009). Finally, among the very late phenomena, acquired at age 6 and later are sentential negation (Wojtecka et al., 2011), exhaustivity in multiple wh-questions (Roeper et al., 2007; Schulz and Roeper, 2011; Schulz, 2015), grammatical gender in Dutch (Blom et al., 2008), and the case marking paradigm in German (Tracy, 1986; Eisenbeiss et al., 2005). Sentential negation and exhaustivity in wh-questions require semantic information and language-external resources. Grammatical gender in Dutch exhibits inconsistent cues for gender values on nouns and requires lexical knowledge (Tsimpli, 2014: 301). Similarly, the case marking paradigm in German exhibits intransparent cues for case marking on determiners, requiring lexical knowledge of the gender of the nouns and of the case suffixes within the tripartite gender system in German.

The few studies testing Tsimpli (2014) timing hypothesis confirm that timing differences result in different patterns for 2L1 and successive bilingual learners. Investigating the effects of age on onset and of input in grammatical gender in Greek (early) and Dutch (very late), Unsworth et al. (2014) found that amount of input was a predictive factor for the pattern attested in both Greek and in Dutch, whereas age of onset could explain the differences between 2L1 and successive bilinguals in Greek, but – as predicted by the timing hypothesis – not in Dutch. Likewise, the age of onset effects found by Meisel (2016) for gender in French, which is acquired early, are in line with the timing hypothesis. Furthermore, in a study with school-aged eL2 children acquiring English with a mean age of onset of 3 years, Chondrogianni and Marinis (2011) found effects of length of exposure rather than of age of onset for the acquisition of the late acquired structures wh-questions and passives. Last, 2L1 and eL2 children have been reported to differ in their comprehension of wh-questions in German, acquired late, with differences being accounted for by length of exposure rather than by age of onset effects (Roesch and Chondrogianni, 2016).

As mentioned at the beginning of Section “Factors Influencing Child Bilingual Acquisition,” differences and parallels may concern acquisition process and patterns, acquisition pace, and acquisition success. The focus of the present study is on pace, i.e., the question of how fast progress on the acquisition of a specific phenomenon is being made, and on success, i.e., the question of whether and at what age a specific phenomenon is acquired. Pace is typically measured quantitatively as the percentage correct in a given task across several time points, and success is typically measured via mastery (e.g., 90% correct) or via emergence of a phenomenon (e.g., first productive occurrence). Note that no matter which measure is chosen results are likely to vary to some degree depending on the specific task used. For example, case marking in German has been reported to be mastered late (Tracy, 1986; Schulz and Tracy, 2011; Schwarze, 2018) but also early (Roesch and Chondrogianni, 2016). Accordingly it is important to consider the specific task when reporting specific ages of mastery. Finally, when considering the effects of timing in L1 acquisition, age of testing is crucial because, as with the effects of age of onset, it necessarily determines whether we can expect monolingual children to have acquired this phenomenon by that age.

### Effects of Language Input and Language Dominance

Children who grow up bilingually receive less input in either language than their monolingual peers (Paradis and Genesee, 1996; Unsworth, 2013a). Nevertheless, simultaneous bilingual children have often been reported to acquire the two languages without delays compared to monolingual children (e.g., Paradis et al., 2011a). Accordingly, roughly half of the monolingual child’s input seems to be sufficient for successful acquisition (Thordardottir, 2010). However, this observation leaves open the issue of how input quantity and quality influence acquisition patterns, pace, and success. Questions of quantity and quality of input have subsequently motivated much bilingualism research (Müller, 1990; De Houwer, 2009). The issue of input quantity is closely tied to questions of language dominance and investigations into which language used with a child is dominant and in which contexts a child receives her input. The issue of input quality is related to children’s parental background, including whether parents (and siblings) are native speakers of the language in question and which socio-economic status or educational background parents have.

Assessment of input factors is difficult, however. They may change over time and they may be connected to child-related factors, such as the child’s language use, language preference, language proficiency, and language output (e.g., Bohman et al., 2010; Schmeißer et al., 2015). Accordingly, the question of how to define reliable measures of quantity and quality of input has recently received increased attention (Paradis et al., 2011b; Unsworth, 2013b; Unsworth et al., 2014; Tuller, 2015; Roesch and Chondrogianni, 2016). Unsworth (2013b) argues for a calculation of cumulative length of exposure, in addition to current amount of exposure, in order to capture the sum of bilingual children’s language exposure over time. Her results on the acquisition of gender in Dutch indicate that both cumulative and current amount of exposure predicted 2L1 children’s performance. However, when 2L1 children were compared with monolinguals in terms of cumulative length of exposure, their scores were as high as (or higher than) the monolinguals.’

Independent of the specific measures used, differences in amount of input have often been shown to affect both bilingual children’s language abilities and the rate at which they acquire various linguistic phenomena relative to monolinguals. For instance, rate of acquisition of vocabulary and morpho-syntax in English/French bilingual children seems to be affected by language input and use (Paradis et al., 2011b). Similarly, in studies of bilingual children a connection was found between amount of exposure and language development for vocabulary and morpho-syntax (Thordardottir, 2010; Hoff et al., 2012). In a similar vein, a study on vocabulary acquisition showed that, provided sufficient exposure to the majority L2 language, children who switched dominance from the L1 to the L2 caught up to their monolinguals peers at an even faster rate than simultaneous bilingual children (Hammer et al., 2008). However, some studies found amount of language input at home in the majority language to be unrelated to children’s language performance (e.g., Chondrogianni and Marinis, 2011, for eL2 learners), one of the reasons being parents’ low proficiency level in the majority language in which the children were tested (Chondrogianni and Marinis, 2011; Paradis, 2011). Similarly, in a study of placement of finite and non-finite verbs in 2L1 children in German, Schmeißer et al. (2015) found that language dominance and grammatical development were not positively related. In short, while it is undisputed that language input and language dominance play an important role for children’s language outcomes, it is far from settled how different language domains are affected and whether input effects are the same in simultaneous bilinguals and in eL2 children.

### Research Questions

The aim of our study is to explore the three factors discussed above – age of onset, timing in L1 acquisition, and language input – by assessing the performance of 2L1 children and eL2 children as well as of monolingual children across early and (very) late phenomena. As for “age of onset” to German, we compare 2L1 children, who are similar to monolingual children in that they have roughly the same age of onset, to eL2 children. This way we are able to shed light on differences and parallels between two groups of child bilingual leaners, which have been argued to constitute distinct acquisition types on theoretical grounds. As for “timing in L1 acquisition,” we compare early, late and very late acquired phenomena to see whether children’s acquisition pace and success differs across differently timed phenomena. The early phenomena under investigation are subject-verb agreement and telicity, the late phenomena are complex sentences and wh-questions, and the very late phenomena are sentential negation and case marking. Timing is considered in relation to the age of testing, which may take place before or after this domain has been mastered by monolingual children. As for the third factor, “language input,” we study language dominance in the 2L1 group to find out whether children who are predominantly exposed to German at home benefit from a higher amount of input in terms of rate of acquisition.

Our first research question (Q1) addresses the effects of age of onset and timing in L1 acquisition and asks how the factors age of onset and timing in L1 acquisition affect the performance of simultaneous bilingual and early second language learning children. More specifically, we assessed the extent to which age of onset (from birth, around age 3) accounts for bilingual children’s performance and whether timing in L1 acquisition (early, late, very late) interacts with age of onset. If bilingual children’s performance is mainly attributed to effects of age of onset, two predictions can be made. First, even relatively small differences in age of onset between the 2L1 (AoO = 3 months in our sample) and the eL2 group (AoO = 35 months in our sample) should result in differences between these two groups, with the 2L1 children performing better than the eL2 children. Exposure of the 2L1 group to the L2 German is longer than exposure of the eL2 group to the L2 German at any given point in time. We hence predict that the advantage of the 2L1 group in terms of an earlier age of onset holds independent of the specific phenomena investigated. Note that this does not imply that eL2 children always lag behind their 2L1 peers: if a specific phenomenon is assessed later in development, *after* the eL2 learners have mastered it, the advantage of the 2L1 over the eL2 learners would no longer be visible – at least in quantitative terms. The second prediction concerns the comparison of 2L1 and monolingual children. If age of onset is the crucial factor in determining children’s performance, simultaneous bilinguals and monolinguals are expected to perform similarly, as length of exposure to German is by definition roughly the same in the two groups, and this pattern should be constant across development. If, however, timing in L1 acquisition interacts with age of onset effects, the patterns of behavior are expected to differ depending on whether the phenomenon in question is acquired early or late or very late. Here we apply Tsimpli (2014) proposal to the acquisition types 2L1 and eL2. Accordingly, 2L1 children are predicted to have an advantage over eL2 children for early acquired phenomena, whereas for late and very late acquired phenomena the two groups are predicted to perform similarly, and different from monolinguals. Again, it should be noted that these patterns may change with age: if testing of an early acquired phenomenon takes place later in development, *after* the eL2 learners have mastered it, the advantage of the 2L1 over the eL2 group will no longer be present, because both will have reached ceiling performance. Crucially, for early acquired phenomena the expected advantage for 2L1 over eL2 children is also predicted by the factor age of onset alone; for (very) late acquired phenomena, however, the factor timing leads to different predictions than the factor age of onset alone. Children’s performance was assessed across six phenomena that varied with regard to their timing in L1 acquisition (early, late, very late). Quantitative measures via the mean scores achieved were used as well as qualitative measures, through assessing whether mastery in that domain was reached. To consider the role of the time of testing in relation to the factor timing in acquisition, data were collected across two test rounds.

Our second research question (Q2) asks whether language dominance affects simultaneous bilingual children’s performance. We restricted the question to the group of 2L1 children, because they are likely to vary with regard to dominance, whereas eL2 learners of German at preschool age are most likely dominant in their L1. Under the assumption that input is especially crucial for the late acquired phenomena, simultaneous bilinguals who are predominantly exposed to German at home are expected to profit from this input and show an advantage over balanced or non-German-dominant simultaneous bilinguals especially in phenomena acquired at age 5 or later. For early acquired phenomena, which we hold to be less influenced by input effects, simultaneous bilingual children should not show differences according to their language dominance.

## Materials and Methods

The data was collected in the course of two research projects, MILA (Grimm and Schulz, 2012) and *cammino* (Schulz et al., 2014). In both projects monolingual and/or bilingual language acquisition in child learners of German was examined in a combined cross-sectional and longitudinal design. The children were recruited between 2008 and 2013 in and around Frankfurt/Main, Germany. The current study reports the results of two test rounds, conducted at the ages of 4;4 years (test round 1) and 5;8 years (test round 2).

### Participants

The sample for test round 1 included 49 monolingual (MON) and 111 bilingual children, all of whom spoke German and one “other” language that differed across children.^2^ Of these children, 37 monolingual and 103 bilingual children participated in test round 2. The bilingual children were further divided into a simultaneous bilingual (2L1) group and a group of early second language (eL2) learners of German according to their age of onset to German. The 2L1 learners had systematic contact to German and the “other” language before 24 months of age. The eL2 learners had an age of onset between 24 and 48 months of age. Background information was collected via a parental questionnaire and telephone interviews with the parents conducted in German or in the parent’s L1. All children visited a German-speaking daycare center.

Subsequent to formal parental consent, children were included in the study if they scored at a standard value of 70 or higher in the non-verbal scales of the K-ABC (Kaufman et al., 2003) at 52.4 months of age (*SD* = 4.7), if there was no assignment to speech-language intervention, and if according to their kindergarten teachers and their parents they showed age-appropriate language development.

Table 1 provides the participant information. Across the three groups children’s parents had a similar socio-economic background, with the exception of the fathers of the 2L1 children, who had a longer school education than the fathers of the eL2 children. Note that the majority of studies uses maternal educational background; we included information on father’s educational background for the sake of completeness. Non-verbal IQ of the monolingual and the eL2 children did not differ, but the 2L1 group had a significantly higher non-verbal IQ than both the monolingual and the eL2 group.^3^

**Table 1:** Mean values and standard deviations for background variables of participants.

	MON			2L1			eL2			
				
	*N*	*M*	*SD*	*N*	*M*	*SD*	*N*	*M*	*SD*	
**Test round 1**										
Child										
Age (months)	*49*	52.2	2.0	*41*	53.0	5.3	*70*	52.3	4.6	
AoO (months)	*49*	0	0	*39*	3.2	7.0	*70*	35.2	4.2	ABC
LoE (months)	*49*	52.2	2.0	*39*	49.6	9.1	*70*	16.5	6.4	BC
Non-verbal IQ	*49*	92.2	11.9	*41*	98.2	12.5	*70*	93.2	12.0	AC
**Test round 2**										
Age (months)	*37*	68.8	1.6	*38*	67.2	7.7	*65*	67.8	6.0	
AoO (months)	*37*	0	0	*36*	3.5	7.2	*65*	35.2	4.4	ABC
LoE (months)	*37*	68.8	1.6	*36*	63.8	10.7	*65*	31.9	7.7	ABC
Mother										
School education (years)	*49*	11.6	1.6	*41*	11.5	1.9	*68*	10.7	2.5	
LoR (years)	*2*	9	1.4	*25*	15.9	7.6	*48*	13.0	7.5	
Father										
School education (years)	*48*	11.2	2.5	*39*	11.7	1.7	*67*	10.5	3.0	C
LoR (years)	*0*	–	–	*23*	16.9	7.2	*46*	14.5	7.2	


*AoO: Age of Onset; LoE: Length of exposure; LoR: Length of residence; A: significant difference between MON and 2L1, B: significant difference between MON and eL2, C: significant difference between 2L1 and eL2.*

#### Monolingual Children (MON)

The monolingual group consisted of 21 girls and 28 boys. All children were born in Germany. In 43/49 cases, children’s parents were also born in Germany, and in six families one parent was born in another country. In all 49 families German was the only home language and the only language the children acquired.

#### Simultaneous-Bilingual Children (2L1)

The 2L1 group consisted of 18 girls and 23 boys. All children except for three were born in Germany. Out of the total of 41 families, in 18 families both parents were born in another country. In 12 out of these 18 families the parents were born in the same foreign country (most often Turkey, Afghanistan, Bosnia/Serbia), and in 6 cases the parents were born in different foreign countries. In 19/41 families one parent was born in Germany and the other parent was born in another country, and in one family both parents were born in Germany. For three families, information was lacking. Children acquired one of 17 different other languages, with Turkish and Russian being the most frequent, spoken by five children each. Age of onset was very homogeneous within the 2L1 group: for the majority of 2L1 children (32/41) age of onset was at birth. For two children, age of onset was after 0 and before 12 months; for seven children age of onset was between 12 and 23 months.^4^

#### Early Second Language Learners (eL2)

The eL2 group consisted of 43 girls and 27 boys. All children except for one were born in Germany. In 49 out of the total of 70 families, both parents were born in another country. In 44/49 families the parents were born in the same foreign country (most frequently Turkey, Afghanistan, Bosnia/Serbia) and in 5/49 families the parents were born in different foreign countries. In 8 families one parent was born in Germany, and in three families, both parents were born in Germany; for 10 families this information was lacking. Children acquired one of 28 different other languages, with Turkish being the most frequent, spoken by 15 children.

Age of onset was homogeneous within the eL2 group as well: 51/70 children had an age of onset between 34 and 40 months, corresponding to the age at which children in Germany typically enter daycare.

#### Language Dominance of the Bilingual Participants

Given the organization of the two projects, a child’s language dominance was determined during test round 1 based on a parental questionnaire targeting language use at home. This resulted in a three-way classification as German-dominant, balanced or non-German-dominant. More specifically, children’s language dominance was calculated as a ratio of non-German and German use by the mother, the father and the child’s siblings. The calculation was based on responses to the following questions: For the languages spoken in their home, each parent was asked *Welche Sprache(n) sprechen Sie mit Ihrem Kind?* “Which languages do you use when talking to your child?” In addition, we asked *Welche Sprache(n) sprechen die Geschwister, wenn sie miteinander sprechen?* “Which language(s) do the siblings use when talking to each other?” For each language that was named one point was awarded. This yielded a maximum score of 3 and a minimum of 0 in each of the two languages. Then for each bilingual child, the ratio of non-German/German was calculated with values ranging between 3 and 0.^5^ If all family members exclusively used the “other” language when speaking with the child, the value was set at 3. The minimum value of 0 was reached if all family members exclusively used German when speaking with the child. A ratio of 1 indicates that family members used both the “other” language and German, when speaking with the child. Children were classified as “non-German-dominant” if the score was >1, as “German-dominant” if the score was <1 and as “balanced” if the score was 1.

Calculation of the ratio for the eL2 group confirms the expectation that at age 4;4 most of the children (51/70) are predominantly exposed to their L1 at home, compared to only 13 balanced and 6 German-dominant eL2 learners. As a result, the factor “language use” was not considered further in the group of eL2 learners. The distribution in the 2L1 group is summarized in Table 2.

**Table 2:** Language dominance in the simultaneous bilingual children.

Language Dominance Ratio	2L1 children (*N* = 41)
**Non-German-dominant (*n* = 11)**	
3	3
2	6
1.5	2
**Balanced (*n* = 12)**	
1	12
**German-dominant (*n* = 18)**	
0.67	11
0.5	6
0.33	1


As can be inferred from Table 2, language dominance in terms of language use is evenly distributed in the group of 2L1children: at age 4;4 11 children of the 2L1 group are dominant in the non-German L1, 11 children are balanced bilinguals, and 18 children are dominant in German. In the sub-group of 32 2L1 children with an age of onset before 12 months, the distribution was the same.^6^ Note that this measure of language dominance in terms of use, assessed via parental questionnaire, differs from more fine-grained measures such as evaluating input quantity and quality via detailed questionnaires or direct assessment; we return to this issue in the discussion.

### Material

Children’s language performance was assessed with the standardized test LiSe-DaZ, administered in German (Schulz and Tracy, 2011), which offers separate norms for monolingual children and for eL2 children. The test was normed on 912 children (609 eL2 children and 303 monolingual children) across eight German states from diverse regions in Germany (see Schulz and Tracy, 2011: 86–87). The majority of the eL2 children spoke Turkish as their first language, followed by Indo-Iranian languages such as Urdu, Kurdish, and Dari and by Russian (Schulz and Tracy, 211: 88). Three subtests assess comprehension of central rule-based language phenomena: Verb meaning (semantics), Wh-questions (syntax, semantics), and Negation (syntax, semantics). Three subscales assess language production via an elicited production task in core areas of morpho-syntax: Complex sentences, Subject-verb agreement, and Case marking. These six scales were further considered for our analyses, for they provide maximum scores, which allow us to calculate mastery of acquisition. Five further sub-scales assess word classes including main verbs, modal and auxiliary verbs, prepositions, focus particles, and subjunctions (morpho-syntax, lexicon). These latter sub-scales were not considered further, as they assess the number of tokens produced, which indicates productivity but are not suitable as a measure of mastery.

The scales Verb meaning (12 test items) and Negation (12 test items) are based on a Truth-value-judgment task that elicits *Yes* or *No* responses. The scale Verb meaning assesses whether children are sensitive to the differences between telic and atelic verbs (see Penner et al., 2003). Contrasting true and false negatives, the scale Negation tests children’s knowledge of sentential negation (see Wojtecka et al., 2011). Using a question-after picture-design, the scale Wh-questions (10 test items) assesses knowledge of argument and adjunct wh-questions by asking children to respond to a wh-question with the correct part of a sentence (see Schulz, 2013). The production scale Complex sentences analyzes the most complex sentence types produced by the child (see Schulz and Schwarze, 2017; Wojtecka, 2018, unpublished) on a scale ranging from 1, indicating utterance of single word utterances only, to 4, indicating use of embedded sentences. A specific level was assigned if the child produced at least three utterances corresponding to that level. The scale Subject-verb-agreement assesses children’s knowledge that in German subject and verb have to agree in number and person (see Schulz and Schwarze, 2017). It is calculated in two steps. First, the number of all utterances containing a subject and verb (sum 1) and the number of all utterances containing a subject and verb with correct subject-verb-agreement (sum 2) are calculated. Second, a ratio is calculated by dividing sum 2 by sum 1, with the maximum score being 1.0. The sub-scale Case marking considers the total number of correctly realized case markings for accusative and dative in object positions and prepositional phrases. The maximum score is 9 (see Schwarze, 2018).

Following Tsimpli (2014) the factor “timing in L1 acquisition” is defined as the age at which specific phenomena are mastered in monolingual acquisition. Based on findings from previous research (see Section “Timing in L1 Acquisition”) the six phenomena studied here can be loosely classified as early, late or very late. However, as noted before age of mastery may vary to some degree depending on the specific task used. For the purposes of the current study we therefore calculated the age of mastery of all phenomena under investigation in a more precise fashion by considering the norming data for monolingual children from the LiSe-DaZ manual. Norming data are available for four age-groups: 3;00–3;11, 4;00–4;11, 5;00–5;11, 6;00–6;11 (for the values, see Schulz and Tracy, 2011: 92, 95–97). The cut-off criterion for mastery was set at 90% (see Unsworth et al., 2014). Regarding the comprehension scales Verb meaning, Wh-questions, and Negation and for the production scale Case marking, we determined the age at which a mean of 90% of the test items were answered correctly. Age of mastery for the remaining two production scales was determined as follows. Regarding the scale Complex sentences, we calculated the age at which the mean score reached 90%. This score corresponds to a raw mean of 3.6 out of 4, with 4 expressing productivity of complex sentences in production. Regarding Subject-verb agreement, we calculated the age at which the correctness rate of subject-verb agreement (i.e., proportion of sentences with correct subject-verb agreement out of sentences with subject and verb) reached 90%. Table 3 summarizes the relevant scales of LiSe-DaZ and the respective age of mastery in the norming sample.

**Table 3:** Scales of LiSe-DaZ, maximum score, size of norming sample, mean raw values (and standard deviations), mean percentage correct, and age of mastery in monolingual acquisition according to the norming sample.

Scale	Maximum score	Size of norming sample	Mean raw value (*SD*)	Mean Percentage correct	Age of mastery in monolingual acquisition
**Comprehension**					
Verb meaning	12	78	10.86 (1.88)	90.50%^∼^	4
Wh-questions	10	63	9.57 (0.64)	95.70%^∼^	6
Negation	12	63	10.89 (1.62)	90.75%^∼^	6
**Production**					
Case marking	9	63	6.21 (1.76)	69.00%^∼^	>6^∗^
Complex sentences	4	73	3.8	95.00%^#^	5
Subject-verb agreement	1	84	0.95 (0.08)	95.00%^+^	3


*^∼^Percentage of test items solved correctly. ^∗^The oldest group of six-year-olds did not reach the 90% criterion. ^#^Percentage of complex sentences out of all sentences. ^+^Percentage of sentences with correct subject-verb agreement.*

In short, according to the norming sample Subject-verb agreement is mastered at age 3 (early), Verb meaning at age 4 (early), Complex sentences at age 5 (late), Wh-questions and Negation at age 6 (late), and case marking after age 6 (very late).

### Procedure and Statistical Analysis

The children were tested by trained student assistants in a quiet room in their kindergartens, and all test sessions were video-recorded. Later analysis was carried out by different research assistants trained on data analysis. As LiSe-DaZ does not provide norms for 2L1 learners, all analyses were based on raw scores. Descriptive statistics were calculated for the study sample characteristics and for the raw scores of the six sub-scales of Lise-DaZ. One-way analyses of variance were used to determine whether the factor Group (monolingual, 2L1, eL2) differed in age, non-verbal IQ, and educational background of the parents. Separate Kruskal–Wallis-Tests (one test for each sub-scale) were used to compare the groups; non-parametric tests were employed because the data were not normally distributed. Significant main effects were followed by pairwise comparisons (Mann–Whitney-*U*-Tests) adjusted for significance by Bonferroni-corrections. Effect sizes were calculated manually as a dividend of the *z*-score (taken from the standard test statistic) and the square root of the overall number of participants: *r* = *z*/√*N* (Field, 2013: 248).

## Results

### Effects of Age of Onset and Timing in Acquisition

First, mean raw values achieved across the two test rounds were computed for the three child groups (see Table 4).^7^ Inspection of the mean values at test round 1 (age 4;4) reveals a uniform pattern of results for all six scales: the monolingual children achieved higher scores than the 2L1 children, which in turn achieved higher scores than the eL2 children. For test round 2 (age 5;8), we observed this pattern in four out of six scales: in the scales Complex sentences, Wh-questions, Negation, and Case marking the monolingual children achieved higher scores than the 2L1 children, which in turn scored higher than the eL2 children. In the scale Subject-verb meaning, the 2L1 group performed like the monolingual group and better than the eL2 group. In the scale Verb meaning the two bilingual groups achieved the same score, which was lower than the score achieved by the monolinguals.

**Table 4:** Mean raw values, standard deviations and mastery for MON, 2L1, and eL2 children in the scales of Lise-DaZ ordered by age of mastery in monolingual acquisition.

Scale	MON			2L1			eL2		
			
	*N*	*M*	*SD*	*N*	*M*	*SD*	*N*	*M*	*SD*
**Test round 1 (age 4;4)**									
Subject-verb agreement (max 1)	*49*	0.97^∗^	0.04	*40*	0.95	0.07	*58*	0.87	0.21
Verb meaning (max 12)	*49*	11.3	1.2	*41*	11.0	1.2	*70*	9.7	2.1
Complex sentences (max 4)	*49*	3.8	0.39	*41*	3.5	0.57	*65*	2.7	0.16
Wh-questions (max 10)	*49*	8.6	1.5	*40*	6.4	2.5	*68*	4.8	2.5
Negation (max 12)	*49*	10.1	1.8	*40*	8.0	2.0	*66*	6.8	2.3
Case marking (max 9)	*49*	4.2	2.3	*41*	2.1	1.8	*68*	1.3	1.6
**Test round 1 (age 5;8)**									
Subject-verb agreement (max 1)	*36*	0.99	0.01	*36*	0.99	0.03	*63*	0.92	0.42
Verb meaning (max 12)	*37*	11.9	0.4	*36*	11.4	0.9	*64*	11.4	0.8
Complex sentences (max 4)	*36*	4.0	0.0	*36*	3.8	0.4	*64*	3.6	0.7
Wh-questions (max 10)	*37*	9.2^#^	0.9	*36*	8.7	1.6	*63*	8.1	1.7
Negation (max 12)	*37*	10.9	1.4	*36*	9.6	2.1	*63*	9.4	1.9
Case marking (max 9)	*36*	5.8	1.9	*36*	3.9	1.9	*64*	3.1	2.0


***^∗^**Shaded cells indicate mastery in this scale. ^#^Mastery is almost reached, the mean value for mastery is 9.57.*

As noted above (see Section “Research Questions”), differences between two learner groups may be absent because both have not acquired the phenomenon under investigation or for the simple reason that testing took place so late in development that both groups, e.g., eL2 learners and 2L1 learners, by the time of testing have reached ceiling performance. To distinguish these two scenarios, we coded for each scale whether the child groups achieved mastery in that domain (i.e., reaching the raw mean value corresponding to the 90% criterion in monolingual acquisition, see Section “Timing in L1 Acquisition”). Table 4 illustrates the results, with shaded cells marking those scales in which the respective child group reached mastery. Across both test rounds, the data for the monolingual group are in line with the ages derived from the norming sample. At T1 the 2L1 group reached mastery in Subject-verb agreement and Verb meaning, and at T2 also in Wh-questions. At T1, the eL2 group reached mastery in none of the six scales, and at T2 only in the scale Verb meaning. Table 5 depicts the results of the inferential statistics.

**Table 5:** Statistical outcome at T1 and T2 (main effect, pairwise comparisons and effect sizes) for the six scales of LiSe-DaZ.

	Main effect			Pairwise comparisons					
		
	Acquisition type			2L1 - MON		2L1 – eL2		eL2 - MON	
				
	*N*	H(2)	*p*	*p*	*r*	*p*	*r*	*p*	*r*
**Test round 1**									
Subject-verb agreement	*147*	19.69	**<0.001**	0.869	0.11	**0.010**	**0.28**	**<0.001**	**0.39**
Verb meaning	*160*	26.49	**<0.001**	0.476	0.15	**0.004**	**0.30**	**<0.001**	**0.46**
Complex sentences	*159*	55.42	**<0.001**	**0.014**	**0.29**	**<0.001**	**0.36**	**<0.001**	**0.68**
Wh-questions	*157*	60.98	**<0.001**	**<0.001**	**0.45**	**0.015**	**0.27**	**<0.001**	**0.72**
Negation	*155*	49.89	**<0.001**	**<0.001**	**0.45**	0.102	0.21	**<0.001**	**0.65**
Case marking	*158*	44.72	**<0.001**	**<0.001**	**0.42**	0.112	0.2	**<0.001**	**0.62**
**Test round 2**									
Subject-verb agreement	135	7.64	**0.022**	0.367	0.18	0.922	0.10	**0.017**	**0.28**
Verb meaning	137	14.04	**0.001**	0.061	0.27	0.819	0.11	**0.001**	**0.37**
Complex sentences	136	19.91	**<0.001**	0.164	0.23	0.076	0.22	**<0.001**	**0.44**
Wh-questions	136	15.70	**<0.001**	0.471	0.17	0.075	0.23	**<0.001**	**0.39**
Negation	136	17.75	**<0.001**	**0.006**	**0.36**	1.00	0.06	**<0.001**	**0.41**
Case marking	136	30.39	**<0.001**	**0.003**	**0.39**	0.242	0.17	**<0.001**	**0.55**


*Significant results are given in boldface.*

Significant main effects were found in test round 1 and in test round 2 for all scales. Turning first to the comparison between 2L1 and eL2 children, pairwise comparisons show that at age 4;4 the 2L1 group performed better than the eL2 group in four out of six scales (Subject-verb agreement, Verb meaning, Complex sentences, Wh-questions), with effect sizes ranging from weak to moderate, and like the eL2 group in the scales Negation and Case marking. Importantly for our argumentation, this parallel between 2L1 and eL2 children cannot be attributed to the eL2 group having already acquired Negation and Case marking by age 4;4, because neither reached the score for mastery in these phenomena. At age 5;8 the pattern is different: the 2L1 group behaved like the eL2 group across all six scales, with all effect sizes being weak. As inspection of mastery in Table 4 shows, this parallel between 2L1 and eL2 children can be partially attributed to the eL2 learners having caught up to their simultaneous bilingual peers, who have mastered Subject-verb agreement, Verb meaning, and Complex sentences by age 5;8. However, this is not true for the scales Wh-questions, Negation and Case marking, which neither the 2L1 nor the eL2 group has mastered at that age. As expected, the eL2 learners performed significantly worse than the monolingual children in all six scales across both test rounds, with effect sizes ranging from moderate to large at test round 1 and from weak to large at test round 2.

Turning to the comparison between simultaneous bilingual and monolingual children, at age 4;4, the 2L1 group performed like the monolingual group in only two scales (Subject-verb agreement and Verb meaning), whereas in four out of six scales (Complex sentences, Wh-questions, Negation, Case marking) the scores of the 2L1 group were significantly lower than those of the monolingual group, with effect sizes ranging between weak and moderate. By age 5;8 the pattern has changed: 2L1 and monolingual children did not differ in four scales (Subject-verb agreement, Verb meaning, Complex sentences, Wh-questions), but in the two scales Negation and Case marking the 2L1 children still performed significantly lower than the monolingual children, with moderate effect sizes. In summary, age of onset alone cannot explain the unique profile exhibited by the changes in the 2L1 group from test round 1 to test round 2.

### Results for Language Dominance

Addressing the second research question of whether language dominance affects the performance of simultaneous bilingual children, we took the three-way classification as German-dominant, balanced and non-German-dominant (see Table 2) as a starting point. Effects of language dominance are most likely to be observed in German-dominant vs. non-German-dominant children, whereas outcome for balanced bilinguals is less clear. Accordingly, we first compared the two extreme groups (German-dominant vs. non-German-dominant) using the Mann–Whitney-*U*-Test in order to detect an influence of language dominance. The results for the six scales across the two test rounds are summarized in Table 6; the results of the inferential statistics are given in Table 7.

**Table 6:** Mean raw values, standard deviations for German-dominant and non-German-dominant 2L1 children in the scales of Lise-DaZ.

	German-dominant			Non-German-dominant		
		
	*N*	*M*	*SD*	*N*	*M*	*SD*
**Test round 1**						
Subject-verb agreement (max 1.0)	*18*	0.95	0.07	*11*	0.96	0.07
Verb meaning (max 12)	*18*	11.3	0.97	*11*	10.6	1.4
Complex sentences (max 4)	*18*	3.4	0.51	*11*	3.5	0.52
Wh-questions (max 10)	*18*	6.2	2.48	*11*	5.9	2.6
Negation (max 12)	*18*	7.3	1.8	*11*	9.2	1.6
Case marking (max 9)	*18*	2.1	1.7	*11*	1.4	1.4
**Test round 2**						
Subject-verb agreement (max 1.0)	*16*	0.98	0.03	*9*	9.9	0.02
Verb meaning (max 12)	*16*	11.5	1.1	*9*	11.2	0.97
Complex sentences (max 4)	*16*	3.8	0.45	*9*	3.9	0.33
Wh-questions (max 10)	*16*	8.6	1.1	*9*	8.9	2.1
Negation (max 12)	*16*	9.6	1.8	*9*	9.9	2.6
Case marking (max 9)	*16*	3.9	1.4	*9*	3.7	2.2


**Table 7:** Statistical outcome for German-dominant vs. non-German-dominant simultaneous bilingual children at test round 1 and test round 2 (Mann–Whitney-*U*-Test) for the scales of LiSe-DaZ.

Scale	*N*	*U*	*z*	*p*	*r*
**Test round 1**					
Subject-verb agreement (max 1.0)	*29*	87.5	-0.567	0.611	0.11
Verb meaning (max 12)	*29*	68.0	-1.49	0.173	0.28
Complex sentences (max 4)	*29*	98.0	-0.052	0.982	0.01
Wh-questions (max 10)	*29*	95.0	-0.182	0.877	0.03
Negation (max 12)	*29*	**41.0**	-2.66	**0.008**	**0.49**
Case marking (max 9)	*29*	77.0	0.310	0.340	0.06
**Test round 2**					
Subject-verb agreement (max 1.0)	*25*	65.0	-0.501	0.718	0.10
Verb meaning (max 12)	*25*	58.0	-1.00	0.452	0.20
Complex sentences (max 4)	*25*	62.0	-0.816	0.598	0.16
Wh-questions (max 10)	*25*	45.5	-1.56	0.136	0.31
Negation (max 12)	*25*	57.5	-0.834	0.419	0.17
Case marking (max 9)	*25*	66.0	-0.346	0.760	0.07


*Significant results are given in boldface.*

As Table 7 illustrates, at age 4;4 German-dominant and non-German-dominant simultaneous bilinguals differed only in the scale Negation, with the German-dominant children performing worse than the non-German-dominant children. In the other five scales, there was no significant effect for the factor group. At age 5;8, German-dominant and non-German-dominant simultaneous bilinguals did not differ in any of the six scales.^8^ A comparison of all three sub-groups of simultaneous bilinguals confirmed this result: at age 4;4 significant main effects were found only for the scale Negation (Kruskal–Wallis-test, H(2) = 6.329, *p*. = 0.042). *Post hoc* comparisons revealed that this effect is due to the difference between German-dominant children and non-German-dominant children (Mann–Whitney-*U*-Test, Bonferroni-adjusted, *p* = 0.039, *r* = 0.39). At age 5;8, no significant group differences were found. In summary, language dominance, measured via the languages spoken at home, did not result in differences between simultaneous bilingual children acquiring German.

### Summary of Main Results

The current study addressed two research questions. Research question (Q2) asked whether language dominance, measured via the languages spoken at home, affects the performance of simultaneous bilingual children. It was answered negatively, as German-dominant and non-German-dominant simultaneous bilinguals were found to not differ in any of the six scales of LiSe-DaZ at age 5;8 and at age 4;4 only differed in the scale Negation (and in the unexpected direction).

Research question (Q1) assessed the extent to which age of onset (from birth, around age 3) accounts for bilingual children’s performance and whether timing in L1 acquisition (early, late, very late) interacts with age of onset. For ease of comparison, the results are summarized in Table 8 for test round 1 (age 4;4 years) and in Table 9 for test round 2 (age 5;8). The symbols in Tables 8 and 9 indicate whether the groups differ statistically (=) and, if so, which group performed significantly better than the other (< or >) (taken from Table 5). The symbols do not express level of performance, i.e., two groups may not differ because they both exhibited ceiling performance or because they both have not yet mastered the domain targeted by this scale. To distinguish these two scenarios, we indicate for each group and scale whether mastery has been reached (taken from Table 4).

**Table 8:** Summary of the pairwise comparisons and group mastery by language domain (test round 1).

Timing in monolingual acquisition	Age of mastery in monolingual acquisition	Language domain	2L1–MON	2L1–eL2	eL2–MON
Early	3	Subject-verb agreement	2L1 = MON	2L1 > eL2	eL2 < MON
	4	Verb meaning	2L1 = MON	2L1 > eL2	eL2 < MON
Late	5	Complex sentences	2L1 < MON	2L1 > eL2	eL2 < MON
	6	Wh-questions	2L1 < MON	2L1 > eL2	eL2 < MON
	6	Negation	2L1 < MON	2L1 = eL2	eL2 < MON
Very late	> 6	Case marking	2L1 < MON	2L1 = eL2	eL2 < MON


*>: sig better than, <: sig worse than, =: n.s., shading indicates that this group has reached mastery in this scale at this test round.*

**Table 9:** Summary of the pairwise comparisons and group mastery by language domain (test round 2).

Timing in monolingual acquisition	Age of mastery in monolingual acquisition	Language domain	2L1–MON	2L1–eL2	eL2–MON
Early	3	Subject-verb agreement	2L1 = MON	2L1 = eL2	eL2 < MON
	4	Verb meaning	2L1 = MON	2L1 = eL2	eL2 < MON
Late	5	Complex sentences	2L1 = MON	2L1 = eL2	eL2 < MON
	6	Wh-questions	2L1 = MON	2L1 = eL2	eL2 < MON
	6	Negation	2L1 < MON	2L1 = eL2	eL2 < MON
Very late	> 6	Case marking	2L1 < MON	2L1 = eL2	eL2 < MON


*>: sig better than, <: sig worse than, =: n.s., shading indicates that this group has reached mastery in this scale at this test round.*

As can be inferred from Table 8, at age 4;4 the 2L1 children perform like their same-aged monolingual peers in Subject-verb agreement and Verb meaning, but worse than the monolinguals in the Complex sentences, Wh-questions, Negation and Case marking. Regarding mastery, by age 4;4 2L1 children, just like their monolingual peers, master Subject-verb agreement and Verb meaning, but different from their monolingual peers have not mastered Complex sentences. Both the 2L1 and the monolingual group have not yet mastered Wh-questions, Negation and Case marking by age 4;4. Moreover, the 2L1 children perform better than the eL2 children in Subject-verb agreement, Verb meaning, Complex sentences and Wh-questions, but like the eL2 children on Negation and Case marking. By age 4;4 the eL2 group has not mastered any of the six phenomena.

As shown in Table 9, at age 5;8 the 2L1 children perform like their same-aged monolingual peers in Subject-verb agreement, Verb meaning, Complex sentences and Wh-questions, but worse than the monolinguals in Negation and Case marking. Regarding mastery, 2L1 children master Subject-verb agreement, Verb meaning and Complex sentences just like their monolingual peers. Unlike their monolingual peers, at age 5;8 2L1 children have not yet mastered Wh-questions and Negation. Both the 2L1 and the monolingual group have not yet mastered Case marking at age 5;8. Moreover, at this age the 2L1 children perform like the eL2 children in all six phenomena. Notably, at 5;8 years, the eL2 group has only mastered Verb meaning.

## Discussion

In this paper we present data from children acquiring German as one of two languages or as the only language. Our goal was to investigate the effects of age of onset (from birth, around age 3) and of timing in L1 acquisition (early, late, very late) in child bilingualism. Three groups of children were included in the study: simultaneous bilingual children (2L1) and early second language learners of German (eL2) as well as monolingual children (MON). 2L1 children had an age of onset of 3 months, and the eL2 children had an age of onset of 35 months. To assess the stability of patterns across development, we collected data at two test rounds: at the age of 4;4 and about 16 months later. Crucially, the three groups did not differ in age (mean age 4;4 at test round 1 and 5;8 at test round 2). To study the factor timing in L1 acquisition, we targeted six phenomena that differ regarding the time at which they are mastered in monolingual acquisition: Subject-verb agreement (early), Verb meaning (early), Complex sentences (late), Wh-questions (late), Negation (late), and Case marking (very late). These phenomena were assessed using the standardized test LiSe-DaZ (Schulz and Tracy, 2011). Use of a standardized test has the advantage that timing in L1 acquisition could be determined by consulting the norming data for the monolingual children, independently from our sample but based on the same tasks as in the current study.

First we wanted to understand how the factors age of onset and timing in L1 acquisition affect the performance of simultaneous bilingual and early second language learning children. More specifically, we assessed the extent to which this difference in age of onset (from birth, around age 3) accounts for children’s performance and whether timing in L1 acquisition interacts with this factor “age of onset.” The differences and parallels found at age 4;4 between 2L1 and monolingual children on the one hand and between 2L1 and eL2 children on the other (see Table 8) point to a unique profile of simultaneous bilingual learners. This cannot be explained by age of onset alone, but by the mediating effect of timing in L1 acquisition on age of onset. First, 2L1 children have more difficulty with later acquired phenomena than the same-aged monolingual children, despite their very similar age of onset and length of exposure at the time of testing. Second, for the (very) late acquired phenomena Negation and Case marking, the difficulties of the 2L1 learners are so prevalent that they perform on a par with eL2 learners. This is remarkable given that the simultaneous bilingual group had an age of onset of about 3 months, resulting in 50 months of exposure to German up to the age at testing, compared to an age of onset of 35 months in the early L2 group, resulting in only 16 months of exposure.

Put differently, at test round 1 the factor age of onset (from birth, around age 3) accounts for the consistent differences between eL2 and monolingual learners and for the observed partial differences between 2L1 and eL2 learners as well as for the observed partial parallels between 2L1 and monolingual learners. Timing in L1 acquisition accounts for the remaining patterns that would otherwise be unexpected: 2L1 learners show parallels to the eL2 learners and differences to the monolingual peers regarding the (very) late acquired phenomena Negation and Case marking. Furthermore, an intermediate pattern regarding Complex sentences and Wh-questions (MON > 2L1 > eL2) suggests that the factor timing in L1 acquisition is sensitive enough to capture the difference between late phenomena to be mastered shortly after the age of testing in L1 acquisition and very late phenomena. Taken together, in (very) late acquired phenomena the factor age of onset is modulated by the factor timing in L1 acquisition.

The data of test round 2, collected 16 months after test round 1 (see Table 9), confirms the proposal suggested for the data of test round 1. At age 5;8 ceiling performance across all three groups was found only for the early acquired phenomenon Verb meaning. This means that for Verb meaning another 16 months of exposure to German were sufficient for the eL2 group to catch up to their simultaneous bilingual and monolingual peers, who had already been at ceiling at test round 1. Parallel to the pattern at test round 1 the 2L1 group performed worse than the monolingual group regarding the (very) late acquired phenomena Negation and Case marking at test round 2. This suggest that the simultaneous bilingual group did not profit from their early age of onset across all phenomena. What is more, in these (very) late acquired phenomena Negation and Case marking, the 2L1 group behaved just like the eL2 group. This finding supports the assumption that the simultaneous bilingual learners’ earlier age of onset and considerably more exposure to German did not result in a general advantage over the early second language learners. For Wh-questions we found an intermediate pattern with the 2L1 group performing on a par with the monolinguals and with the eL2 group. These results clearly indicate that age of onset alone cannot account for the acquisition patterns found in our data. Timing in L1 acquisition contributes substantially to accounting for the observed parallels and differences between 2L1 and eL2 children on the one hand and 2L1 and monolingual children on the other. Note that the role of timing in L1 acquisition is most clearly visible in the (very) late acquired phenomena Complex sentences, Wh-questions, Negation, and Case marking, which were tested *before* their mastery in monolingual children.

Our findings on age of onset effects in eL2 acquisition are in line with the research discussed earlier (see Section “Age of Onset Effects in Child Bilingualism”). Compared to monolingual children eL2 learners show a delay in rate of acquisition. Moreover, our data show that “catching up” does not necessarily happen at the same pace. Although both verb meaning and subject-verb agreement are acquired early, by age 5;8 eL2 children caught up to their monolinguals peers in the former but not in the latter domain.

Crucially, our results indicate that simultaneous bilingual children do not consistently show the same acquisition rate and age of mastery as their monolingual peers. This contrasts with previous findings by Tracy and colleagues (Gawlitzek-Maiwald and Tracy, 1996; Tracy, 1995, unpublished) but confirms studies pointing to partial delays exhibited by 2L1 children, at least in one of their languages (Gathercole and Thomas, 2009; Bialystok and Hakuta, 2010). More specifically, our findings provide further evidence that age of onset effects are modulated by effects of timing of phenomena in L1 acquisition, as proposed by Tsimpli (2014). The design of our study enabled us to advance the debate on effects of timing in L1 acquisition in several ways. First, we assessed in the same groups of children a variety of phenomena in morpho-syntax and semantics differing with regard to timing in L1 acquisition. This permitted us to explore multiple asymmetries across domains. Second, using a standardized test (LiSe-DaZ, Schulz and Tracy, 2011) we could derive the ages of mastery directly from the monolingual norming sample. The resulting classification as early, late or very late acquired – based on the same tasks we employed with the participants in the current study – has the advantage of being well-defined and specific to the task. This freed us from the necessity of inferring information about age of mastery from the literature, which is likely to vary with the task used (see, for instance, Roesch and Chondrogianni, 2016, who argue based on production studies that case is early acquired in German). Third, since the children were assessed twice over an interval of 16 months, we could explore how parallels and differences between simultaneous bilingual children, early second language learners and monolingual children develop over time.

By comparing 2L1 and monolingual children as well as 2L1 and eL2 children, the present study extends previous research on late acquired phenomena such as gender (for Welsh: Gathercole and Thomas, 2009; for Dutch: Unsworth et al., 2014), comprehension of passives (Chondrogianni and Marinis, 2011; Armon-Lotem et al., 2015) and comprehension of wh-questions (Chondrogianni and Marinis, 2011; Roesch and Chondrogianni, 2016). In line with those studies, for the (very) late acquired phenomena sentential negation and case marking we did not find an effect of age of onset for the 2L1 group, i.e., we did not find an advantage of the 2L1 over the eL2 children. Rather, the 2L1 group, with an age of onset of about 3 months, performed as low as the eL2 group, who had an age of onset of about 35 months, on negation and case marking at both ages 4;4 and 5;8. Furthermore, in line with the timing hypothesis, the four late or very late acquired phenomena complex sentences, wh-questions, sentential negation, and case marking were all found to pose difficulties for the simultaneous bilingual children. Despite their similar age of onset, the 2L1 children had significantly more difficulty than the monolingual children in these four phenomena at age 4;4. Notably, for negation and case marking this difference between 2L1 and monolingual children was still observable at age 5;8.

The second question we asked was whether language dominance influences bilingual children’s performance across language domains. We addressed this question by looking more closely into the language dominance of the 2L1 group measured as language use at home; the majority of the eL2 children was dominant in the “other” language, as expected. Based on a parental questionnaire all 2L1 children were categorized as German-dominant (*n* = 18), balanced (*n* = 12) or non-German-dominant (*n* = 11), depending on whether father, mother and siblings used German and/or the “other” language when speaking with the child. Except for negation, which was in fact easier for the non-German-dominant children than for the other two subgroups, there were no significant differences between the three groups at either age 4;4 or age 5;8.^9^ This result was as expected for early acquired phenomena. Regarding late acquired phenomena the lack of advantage for German-dominant over non-German dominant simultaneous bilinguals was unexpected.

This missing effect of language dominance, measured as the dominant language used at home, for the simultaneous bilingual children in our study contrasts with studies that reported an effect of input factors on children’s performance, such as amount of input (for vocabulary and morpho-syntax: Thordardottir, 2010; Paradis et al., 2011b; Hoff et al., 2012; for gender in Dutch: Unsworth, 2013b) and dominance (for vocabulary: Hammer et al., 2008). Our results agree with the findings by Chondrogianni and Marinis (2011) on eL2 children, who did not find amount of language input at home in the majority language to be related to children’s language performance. The authors argue that their finding may be related to parents’ low proficiency level in the majority language that the children were tested in (see also Paradis, 2011; Paradis and Jia, 2017). In our case, use of German as the dominant home language with the child did not facilitate simultaneous bilingual children’s performance in either the early or the late acquired phenomena. This finding points to the general issue of how to assess the quality and native-likeness of the parental input that children are exposed to. Given that parental fluency has been found to be modulated by parental education (Chondrogianni and Marinis, 2011; Hoff et al., 2012), we may ask whether parental fluency and parental education in both children groups differed. While we could not collect data on the parents’ level of proficiency or fluency to address this question directly, we do have information about parents’ educational background. This data indicates that the parents of the German-dominant and non-German-dominant 2L1 children had a similar educational background (see Footnote 8). It is hence likely that parental fluency in the tested majority language in both groups was similar as well. If parents’ proficiency level in their L2 was low, then this factor may have overridden potential effects of language dominance, as the findings Chondrogianni and Marinis (2011) would suggest.

Alternatively, the presence of another developing language system may cause the language learner to weigh the two systems, which leads to the unique profile of simultaneous bilingual language learners. We agree with Tsimpli (2014) that timing of a structure in L1 acquisition is relevant, with late acquired phenomena being more difficult for 2L1 children than for their monolingual peers, making the 2L1 children look like early second language learners. However, rather than attributing these patterns to overall effects of input (e.g., in terms of length of exposure), we suggest switching perspective and looking at the acquisition task from the learner’s point of view in order to capture the role of the input to the child in a more fine-grained way. We call this concept the learner’s “internal need for time” to acquire a structure or property. More specifically, we propose that the amount of internal time needed is determined by two factors that have figured prominently in recent acquisition research: the “complexity” of the structure or property to be acquired and the “cross-linguistic robustness” of the phenomenon as well as the rule governing it. As noted before (Section “Timing in L1 Acquisition”), formal complexity may refer to how much idiosyncracy and irregularity is involved in a construction (see Culicover, 2014), which may or not may not align with the distinction between core and non-core. Cross-linguistic robustness refers to the issue of how much language-specific variation a construction or its interpretation exhibits (see e.g., the COST Action A 33 on cross-linguistically robust stages of children’s linguistic performance). Put differently, we suggest that when complexity and cross-linguistic robustness are considered, the role of age of onset and language input for bilingual children’s rate and success of acquisition can be addressed more comprehensively. A case in point are phenomena at the semantic-syntactic interface such as sentential negation tested in the current study. Sentential negation involves a complex mapping of syntactic position and meaning and is acquired late. Notably, the interpretation rules are assumed to be cross-linguistically the same, which arguably follows from the general assumption that well-formedness conditions on semantic representations are universal (see also Tsimpli, 2014: 296). Acquisition of sentential negation in bilingual children should hence be unsusceptible to differences in amount of input. This would also be compatible with the finding that in this scale German-dominant children actually performed worse than non-German-dominant children. The same reasoning holds for example for exhaustivity in single and multiple wh-questions, which is acquired late and seems to follow universal interpretation rules (Schulz, 2015). The situation is different for the German case marking paradigm tested in the current study. Because of its complex, intransparent form-function mapping described above, it is acquired very late. However, unlike sentential negation the system of case marking widely varies across languages, just like grammatical gender discussed above. Acquisition of these phenomena, which underlie language-specific licensing rules, should be more sensitive to input effects. This is because, in addition to the time needed to weigh the two developing language systems, the more input the learner receives in the target-language the faster she can make the necessary language-specific choices. This proposal makes specific predictions based on cross-linguistic robustness that need to be tested in future studies.^10^

In this study we explored the interaction of age of onset (from birth, around age 3) and timing in L1 acquisition across different language domains and across development in two different groups of bilingual children that have been argued to constitute distinct acquisition types on theoretical grounds. Whereas chronological age and a number of external variables were controlled for, it was not possible to clearly dissociate age of onset effects from effects of length of exposure. In future research, a group of eL2 children who have the same length of exposure as the 2L1 group could be included as well as a bilingual sample in which age of onset is varied. Due to the set-up of the project, bilingual children acquired many different “other” languages; hence specific effects of the L1 could not be studied. Furthermore, as our small-scale longitudinal design revealed, time of testing plays an important role for detecting effects of both age of onset and timing in L1 acquisition. In future studies, the longitudinal aspect could be expanded, also assessing a wider range of late acquired phenomena. Use of a standardized test allowed us to assess a number of different phenomena and to derive precise ages of mastery. Future experimental studies targeting the phenomena in more detail could shed light on the variation within a scale, e.g., dative case being acquired later than accusative case. Our finding that language dominance did not affect 2L1 children’s performance could be followed up with studies employing more fine-grained measures of dominance including assessment of parental language proficiency. Finally, 2L1 children were found to perform better on the non-verbal IQ test than both the monolingual and the eL2 children. However, the role of non-verbal IQ was limited to few subscales and did not reveal any systematic pattern. This result is in line with previous studies (Schulz and Tracy, 2011: 109; Wojtecka, 2018, unpublished) that found only very few, weak correlations between non-verbal IQ and performance on the LiSe-DaZ subscales. Future studies could explore this factor in more detail for the group of 2L1 children.

## Conclusion

Our findings indicate that in the context of children acquiring German, timing in L1 acquisition is an important factor in child bilingual acquisition, interacting with effects of age of onset, even for learners with an initial exposure to German before age four. Whereas bilingual children’s performance in early acquired phenomena could be explained by age of onset effects alone, only the impact of timing could account for pace and success of acquisition in late acquired phenomena. The observation that the factor language dominance for the simultaneous bilingual group, measured as language use at home, did not affect children’s rate of acquisition, led us to propose an alternative concept to capture the apparent role of input: the learner’s need for time to master a phenomenon, which is determined by its complexity and by its cross-linguistic robustness.

## Ethics Statement

The study was based on data from two projects (MILA and cammino), both conducted in accordance with the Declaration of Helsinki; informed written consent was obtained from the parents of all participants, including publication of the results. The project MILA (PI: PS) was approved by the ethics committee of the German Psychological Association (DGPs) on March 12, 2009. The project cammino (PIs: PS, AG) was approved by the ethics committee of the Department of Psychology and Sports Sciences of Goethe University on June 24, 2011.

## Author Contributions

PS contributed conception and design of the study and wrote Sections “Introduction,” “Factors Influencing Child Bilingual Acquisition,” “Results,” and “Discussion.” AG was responsible for managing data compilation and performing the statistical analysis, and wrote Section “Materials and Methods.” Both authors contributed to manuscript revision of the text and read and approved the submitted version.

## Conflict of Interest Statement

The authors declare that the research was conducted in the absence of any commercial or financial relationships that could be construed as a potential conflict of interest.

## Footnotes

1 The question of whether the distinction between core and non-core can account for the earliness and lateness of all phenomena across languages is beyond the scope of the present study.

2 The data at test round 1 were analyzed in Grimm and Schulz (2016) with a focus on language assessment. Due to the many different L1’s attested in our sample, the role of the L1 is not considered further in the current study. In previous studies we did not find an effect of the L1 for eL2 children’s performance in LiSe-DaZ scales (Schulz and Tracy, 2011; Schwarze, 2018; Wojtecka, 2018, unpublished). See also Tracy and Lemke (2012) and Tracy and Thoma (2009), who report that morpho-syntactic development was independent of the child’s L1.

3 We return to the factor non-verbal IQ in the results (see footnote 7).

4 A re-analysis of the subgroup of the 34 2L1 children with an age of onset before 12 months did not change the results; this holds for the participant variables (chronological age, IQ) as well as for the test results.

5 For single parents, the calculation of the ratio did not change; the maximum value in this case was 2.

6 Of these 15 (47%) were German-dominant, 11 (34%) balanced bilinguals and 6 (19%) non-German-dominant. Of the 7 children with an age of onset between 12 and 23 months, 3 (43%) children were German-dominant and 4 (57%) were non-German-dominant. Correlation between age of onset and language dominance was marginally significant, *χ*^2^(2) = 5.728, *p* = 0.057, *φ* = 0.383.

7 Recall that there were significant differences between 2L1 and eL2 children with regard to paternal school education and between MON and 2L1 children as well as between 2L1 and eL2 children regarding non-verbal IQ (see Table 1). To explore the potential effect of these differences for the group results, additional statistical analyses were employed. Since differences were restricted to some of the groups and the data was not treated as a binary variable, multiple linear regressions comparing MON and 2L1 children and 2L1 and eL2 children were performed. There was no effect of paternal school education for the 2L1 and eL2 children. A significant effect of non-verbal IQ was found in only 7 out of 24 cases: in three cases for the MON and 2L1 children (Subject-verb agreement at T1, Verb meaning and Negation at T2) and in four cases for the 2L1 and eL2 children (Verb meaning at T1 and T2, Wh-questions and Case marking at T1). These results suggest that paternal education and non-verbal IQ did not systematically affect children’s performance.

8 Unlike in other studies, in our sample of 2L1 children, German-dominant and non-German-dominant children did not differ regarding the educational background of parents (mothers: *t*(27) = –0.874; *p* = 0.390; fathers: *t*(26) = –0.470; *p* = 0.642).

9 As pointed out by one of the reviewers, it comes as a surprise that German-dominant children were found to perform worse than non-German-dominant children at all. Note that this effect was present in one subtest, Negation, and at one test round only. We speculate that the way sentential negation was tested in the present study draws on comprehension abilities that are similar cross-linguistically, rendering dominance in the language of testing less important. Further studies are needed to explore this possibility.

10 Thanks to C. Hamann for pointing out to us that lateness of negation could also be language-specific. The interplay of syntactic position and meaning may be particularly difficult for German negation because of its less obvious scope properties. Further studies are needed to clarify this point.
